# Learnings from Thailand in building strong surveillance for malaria elimination

**DOI:** 10.1038/s41467-022-30267-x

**Published:** 2022-05-13

**Authors:** Jui A. Shah

**Affiliations:** RTI International, Unit 406, 208 Wireless Road, Lumpini, Pathumwan, Bangkok, 10330 Thailand

**Keywords:** Policy and public health in microbiology, Parasitology, Malaria

## Abstract

On the cusp of *Plasmodium falciparum* (*Pf*) elimination, Thailand is accelerating towards zero malaria by 2024. This commentary reviews the heart of its success—effective surveillance—and what else may be needed to reach zero on time.

Thailand aims to eliminate malaria by 2024, following China’s malaria elimination certification in 2021 and Sri Lanka’s in 2016. The country reported just 2893 malaria cases last year, down from 24,332 in 2015, mirroring broader progress across the Greater Mekong Subregion (GMS). Regional collaboration has supported Thailand’s success, as malaria epidemiology is heavily influenced by population mobility across forested areas along international borders. Thailand has joined a cohort of 25 countries (dubbed E-2025) with the potential to halt malaria transmission by 2025^1^. To do so, it plans to build on longstanding success factors—robust case-based surveillance informing decentralized action—complemented by new approaches.

## Designing surveillance for elimination

A functional surveillance system is crucial for improving malaria services and for measuring progress toward elimination goals, as affirmed in the World Health Organization’s Global Technical Strategy for malaria, which named surveillance as a core intervention^2^. Malaria free status is only bestowed upon countries that can affirm, beyond reasonable doubt, the absence of local transmission for three years. These criteria necessitate a surveillance system that generates complete, reliable, and timely data suitable for action.

Thailand’s National Malaria Elimination Strategy 2017–2026 (NMES) reoriented the malaria control program into an elimination program centered on upgraded surveillance. Thus, in 2017, Thailand adapted the 1-3-7 strategy from China to prioritize timely, evidence-based action^3, 4^. For each confirmed malaria case, notification occurs within 1 day, case classification within 3 days, and local response within 7 days. The resulting data guide district teams to conduct reactive case detection, coordinate across sites for patients with travel history, and identify vector control targets to further inhibit transmission. Adherence to 1-3-7 protocols exceeded 80% within the first few years^3^, and preliminary results suggest the strategy is effectively driving elimination by encouraging rapid response.

The NMES also introduced subvillage-level stratification of “foci” based on past or current malaria transmission. Stratification is an essential tool for considering heterogeneity in epidemiology, geography, and health systems to optimize malaria interventions and the use of resources. Utilizing foci as the unit of measurement results in remarkably detailed information for Thailand’s interventions and analyses. This granularity, a hallmark of malaria elimination programs, also supports a culture of data use by making information relevant and actionable for local jurisdictions.

Simultaneously, Thailand launched integrated drug efficacy surveillance (iDES) to address multidrug-resistant parasites, which have made elimination challenging and increasingly urgent^5–7^. As part of routine malaria care, treated patients are tracked for up to 90 days to confirm parasite clearance and monitor antimalarial drug efficacy^8^. In 2019, after iDES results, triangulated with external findings, documented subpar piperaquine performance, the National Drug Policy Committee approved pyronaridine-artesunate as a new first-line treatment for two provinces^7, 9^.

These surveillance data are collated into one malaria information system, alongside data on intervention coverage, entomology, and finances^10^. Malaria officers review data to identify unusual results, adjust programming, and inform operational research and strategic planning to propel continued progress toward elimination.

## Cross-continental trends in surveillance

Like Thailand, a growing number of national malaria programs across sub-Saharan Africa (SSA) are facing multiple transmission settings within country boundaries. To remain relevant, the surveillance system and in-country expertise must evolve to meet changing data needs. For many countries in SSA, this will begin with a transition from aggregate to case-based reporting. But Thailand’s experience shows that continual subsequent system refinements are needed. Additional tools, such as interactive dashboards and mobile applications, can promote appropriately decentralized data review and decision-making in a heterogeneous context but may also require revisions. There is limited understanding about the cost of maintaining these iterations of effective malaria surveillance from control to elimination^11^.

There is newfound demand for drug efficacy monitoring following the emergence of partial artemisinin resistance in *Pf* parasites in Uganda^12^ and Rwanda^13^. As similar trends may have put pressure on partner drugs such as piperaquine in the GMS^14^, these findings may threaten a range of antimalarial formulations in SSA. GMS countries have substantial experience to share, developed through regional collaboration. Active participation in regional networks on drug efficacy surveillance, which is inherently a cross-border issue, will support data sharing, research on potential causes, and development of symbiotic countries' strategies, as well as maximize available resources in SSA.

## Reaching and maintaining zero malaria

The legacy of Thailand’s success is a complex map of remaining cases clustered in hard-to-reach areas and populations. As the interventions that enabled this success may not suffice to reach zero malaria, the country is always considering new approaches.

As in many elimination settings, Thailand has seen *Pf* succumb to its efforts, resulting in a proportional rise in *Plasmodium vivax (Pv)*, which was responsible for 94% of cases last year^15^. Since *Pv* can relapse, causing iterant episodes of malaria^16^, interrupting transmission is insufficient for elimination. Thailand has been successfully using 14-day primaquine for radical treatment to prevent relapse and is exploring the feasibility to safely introduce shorter course tafenoquine to improve treatment adherence^17^.

Thailand’s primary malaria vectors show preference for outdoor biting, limiting the effectiveness of interventions like insecticide-treated nets and indoor residual spraying^18^. This challenge is exacerbated by long hours spent outdoors playing, traveling, or working by populations like school-aged children, forest goers, and seasonal workers. Promising research that could support Thailand’s elimination goals includes chemoprophylaxis for forest goers^19^, topical microbial repellants^20^, and mass drug administration with endectocides like ivermectin^21, 22^.

Prevention of re-establishment (POR) planning is a requirement for malaria elimination and a key component of Thailand’s strategy to sustain fragile gains. Provincial POR plans will utilize an expanded stratification, comprising epidemiological and environmental receptivity and vulnerability, to outline actions that prevent local transmission if cases are identified. These actions will rely on a broad workforce as part of Thailand’s gradual integration of its vertical malaria program into the general health system.

Vital to the success of both POR and elimination will be the retention of high-quality surveillance, despite an inevitably reduced central malaria team. In addition to building technical capacity, investing in data quality and interoperability can simplify fundamental analyses for a general health staff with competing priorities. The comprehensiveness of Thailand’s surveillance approaches is admirable; however, in a future scenario of very few, dispersed malaria cases, nimbler strategies that balance risk of transmission with available resources could be beneficial.

There is a reciprocal relationship between malaria burden and surveillance needs, resulting in increasing costs per case as countries approach elimination. Unfortunately, where malaria decreases, risk perception may follow, and it may become harder to rally political, financial, and popular support for malaria programming. To fully fund the NMES, Thailand advocated for domestic resources to complement external funds^23^. Since then, funding partners are increasingly harmonizing their resources. In tandem, Thailand is encouraging subnational units to prioritize malaria in their local budgets and engage in malaria programming.

Finally, a crucial factor for success will be continued leadership that prioritizes compassion and equity. Malaria patients may live on the fringes of society or geography, and elimination will require flexible and safe ways to reach the displaced, underemployed, and unreached. Policy and resilience are strengthened by a plurality of perspectives, so engaging women, youth, and minority communities in decision-making may spark the creativity needed in last-mile endeavors. Thailand is setting a notable example, with women at the helm of the Division of Vector Borne Diseases and several of its subunits.

Although at the global level, momentum against malaria has slowed, Thailand exemplifies that for some countries—including several in the GMS—crossing the malaria elimination finish line is a reality within view.

### Reporting summary

Further information on research design is available in the Nature Research Reporting Summary linked to this article.

## Supplementary information

Reporting summary
